# Answer to a Friend on Organising the Medical Association

**Published:** 1852-08

**Authors:** Samuel Jackson

**Affiliations:** Formerly of Northumberland


*Answer to a Friend on organising the Medical Association.* By SAMUEL JACKSON, M. D., formerly of Northumberland.

MESSRS. EDITORS,—One of your correspondents thinks that the exclusion of *permanent members* from the *meetings* of the American Medical Association, would be unjust to them, and taking from them a privilege they have legitimately earned and one that has been accorded to them by the constitution under which they have acted. There is a show of truth in this reasoning, and we shall therefore inquire, by what means our projected exclusion may be justified.

All human associations are kept together, whether in politics, religion, or science, by mutual concessions, and often by injustice similar to that which is now complained of. In the forming of any constitution or code of laws, it is impossible to attain, at once, what is just and best and ought to be permanent. In the year 1838, the Constitution of Pennsylvania underwent a thorough scrutiny and the good old method of appointing judges whether supreme or not supreme, was confirmed and supposed to be established; therefore men of learning and talents, I hope some of them were such, quit the Bar and accepted appointments from the Governor, as they believed, for life. A few years roll by, the people determine to make their own appointments, and behold these men are deposed, I think that in their own *beautiful* language they say *ousted*.

There is nothing *perfect* of man's creating, hence there ought to be in all human associations, a generous spirit of accommodation, which indeed is the very ground-work of all society and of all government. Now where can we hope to find this if it be not found in the Medical Profession ? Above all men in this world, physicians ought to be ready to accommodate and to forget themselves in the promotion of good works. The moment a young man enters the threshold of medicine, he makes a great sacrifice on the altar of benevolence—*he denies himself and takes up his cross.* We may say of ourselves what Shylock says of the Jews, " sufferance is the badge of our nation." We must not therefore keep up a contention for our ultimate rights ; to demand the last farthing is ignoble and ungenerous—*de minimis non curat Prætor*, says the Roman code.

But if it appear to a majority of the Association, that the exclusion would be an injustice, let it be only prospective ; let the existing members enjoy their privilege ; it can last for a few years only ; and during this time the meetings will not be so large as to be encumbered by the few that will attend. We are trying to legislate for the future and not for the present generation.

But your ingenious correspondent finds yet another objection to the exclusion bill—*committees and officers are often continued from year to year.* This plea may be very easily disposed of. The same difficulty exists in our Pennsylvania State Society and yet we never found any trouble therefrom ; for when the County Societies elect delegates to the State Society, they are careful to choose, among others, those who are officers and committee men. This ought however to be obligatory on them, and I had intended to propose at the last meeting of the State Society, a constitutional amendment to this effect ; but there seemed to be no time to spare for things not pressing. Such an amendment might be easily introduced into the constitution of the Association ; or it might be decreed that all committee men and officers are *ex officio* delegates to the next meeting.

Your correspondent thinks that it " rests with the proposers of the innovation to justify it if they can." This is sound doctrine and we adopt it without hesitation. We are not much conversant with human affairs and therefore speak with great diffidence ; but as far as our knowledge goes, the admission of *permanent members* is an anomaly in human legislation. Does it exist in any other association whether religious, political, or scientific ? What interminable wrangling would there not be in congress, state legislatures, in religious conventions, if the rule obtained! and do you suppose that our physicians are free from the troublesome vice of obtrusive loquacity? If this rule of admission be continued, the time will come and will soon overtake us, when no house will hold both them and the delegates; no voice be heard by the vast assembly; no time will suffice for their ambitious *demonstrations*—I beg pardon for using this word in our native sense.

Another objection is, that some *permanent members* may deteriorate and become totally unworthy of the privilege; they may become such as you would not delegate, such as you would be ashamed to meet. But says your correspondent—"the Association has the power to expel members who have proved themselves really unworthy." Now I should like to know where that power is given. But suppose the constitution were amended to this effect, what a pretty piece of business it would occasion at a meeting of the great Association, where there ought to be nothing unworthy of the *pure physician*, nothing but harmony, good feeling, and love! What, prosecute in this sacred place a poor unfortunate brother for stealing your patient or selling quack medicines! fie, fie. "Don't wash your dirty linen in public."

In what your correspondent calls his "real argument of importance," he makes a most capital blunder. He deprecates "the abolition of permanent membership," and says, "that if those appointed delegates one year, cease with that year to be members of the Association," there would be a difficulty in the appointing of special committees. Now this deprecated abolition of permanent membership has never been proposed. Our plan is, on the contrary, to make every respectable physician in the United States a permanent member as quickly as possible. Look to page seven of our little discourse before the Medical Society and you will find these words—"the privilege granted to what are called permanent members of attending all subsequent meetings and partaking in the debates, is a monstrous anomaly." Now you see that it is not permanent *membership* that we would abolish, but the permanent *privilege* of attending the meetings and making speeches to obstruct the proceedings.

Your correspondent says, "these privileges (of being elected delegates) should be made to rotate amongst all whose characters and attainments are worthy of a place in the great American fellowship." Very well said—*si sic omnia dixisset.* Of this the County and State Societies will doubtless be very careful, but you must not expect them to prove infallible. Their respect for Professors will always appear, and a full proportion of them will always be elected. It has been proposed, more cunningly than wisely, to allow those schools which live up to a certain *beau-ideal*, to send delegates; and the excellent and authoritative author of this project thinks, that the schools will thus be goaded to a more faithful discharge of their duty. The *good* workings of this began at once, even at Richmond. At the first mention of it, the University of Virginia took fire as by lightning, and exploded, under the direction of a master engineer, with such violence as to frighten a majority out of their wits and to obfuscate others with sand and dust in their eyes: hence they received the said University on its own terms. So much for the first experiment—time will tell the rest, if the new plan should be adopted. Schools will come in from at least three of the four cardinal winds, and the question will arise every year, whether this and that shall be admitted or rejected. Let us advise the anxious schools to bring forward their case in the afternoon, when the members have been saturated with good dinners; for this is the time, according to metaphysicians, to ask a favor. Physicians like other mortals, are fond of good cheer; and that they feel the good effects thereof, let them testify for themselves. At the meeting in Charleston they devoted a marble tablet to commemorate their satisfaction and their "grateful sense of the hospitalities and of the numerous delicate attentions" received: and so profoundly were they impressed with the good and benevolent effects of a full stomach that they *unanimously* resolved to hold the "hospitalities and delicate attentions" among their "most pleasing recollections as long as life and thought shall endure." Now mark, this was done after dinner. Voltaire thinks that a comfortable evacuation has an equally benevolent effect—we leave it to the Association to decide. The author of the above potent resolution, had just offered a pious amendment to the constitution by inserting the words—"in reliance on Divine guidance and support;" but you see it required none of this extraneous *support* to enable him to recollect in the awful hour of death, the good cheer of Charleston.

At Richmond too, the fascinations of the table received their merited praise, nor did they fail of producing their wonted effects. If the Association were so ungrateful as to decree no marble memorial, nor yet to promise some pleasing recollections of them *in the agonies of death*, some members, more sensible to the blessings they enjoyed, were thrown thereby into such an extatic state as to dance most lustily to the orgies of the Virginian Bacchus, as though they would sweat off the poison of the tarantula. Like Tam O'Shanter's warlocks and witches—

Hornpipes an' jigs, strathspeys an' reels
Put life an' metal i' their heels.

Now Messrs. Editors I beg pardon for this long digression, pardon too of my friend Hartshorne for leaving him so long; and if you consider it an intolerable evil, I would recommend a method of deriving from it some compensative good. It was an example in my first grammar, that it is an exceedingly good thing to profit by another's madness—*Optimum est alienâ frui insaniâ.* Whoever then among physicians is afflicted with low spirits, vapors, blues, cold feet, a feeble circulation of blood and spirits, let them go to New York next May, prepare their stomach in the manner of the venerated Ancients, and then put on pumps.

Now for this truly important advice we expect no other reward than to be favored with their "most pleasing recollections so long as life and thought shall endure;" and that when death hovers over them with his sable wings—*cum mors atris circumvolat alis*—they will not forget their humble adviser and friend.

